# Visual Screening of Genetic Polymorphisms in *eae* Gene of *Escherichia coli* O157:H7 with Single-Nucleotide Resolution by ARMS-PCR-Mediated Lateral Flow Strip

**DOI:** 10.3390/s26030907

**Published:** 2026-01-30

**Authors:** Noor Fatima, Liangliang Jiang, Siying Sun, Li Yao, Yubo Peng, Daoli Chen, Wei Chen

**Affiliations:** 1 Engineering Research Center of Bio-Process, Ministry of Education, Key Laboratory for Agricultural Products Modern Processing of Anhui Province, School of Food and Biological Engineering, Intelligent Manufacturing Institute, Hefei University of Technology, Hefei 230009, China; nf501324@gmail.com (N.F.); 18975657836@163.com (S.S.); yb0403p@163.com (Y.P.); 2 Health Supervision Institute, Ma’anshan Center for Disease Control and Prevention, Ma’anshan 243000, China; jll505@163.com; 3 School of Food and Bioengineering, Changsha University of Science & Technology, Changsha 410114, China; ybl200999@126.com

**Keywords:** *E. coli* O157:H7, *eae* gene, single nucleotide polymorphism (SNP), lateral flow strips (LFS), direct visual detection, ARMS-PCR, food born pathogen

## Abstract

Development of rapid, precise and fieldable detection methods for foodborne pathogens is one of the essential requirements in food safety and public health. In this research, the single-nucleotide polymorphisms (SNPs) in the *eae* gene of *Escherichia coli* O157:H7 are well visually identified with the designed amplification refractory mutation system–polymerase chain reaction (ARMS-PCR) mediated lateral flow strip (LFS). Allele-specific primers were designed and optimized to discriminate the mutant-type genes from wild-type genes with single-nucleotide resolution in a simple visual format. The single-nucleotide variation in the *eae* gene could be easily differentiated by the observation of an optical signal on the T line of the LFS without any devices. Assay performance results show that it has a high sensitivity and specificity with the single-nucleotide differentiation ratio as low as 0.1%. This genetic polymorphisms screening performance could enumerate complex genetic variation into a simple and direct yes/no readout, highlighting the ultra-easy SNP screening mode and the simplicity of the result output for practical applications. This ARMS-PCR mediated LFS offers a straightforward, swift, and economical strategy for SNP identification with great potential for use in evolution of bacterial resistance genes and viral evolution under different environmental stresses.

## 1. Introduction

Bacterial infections, a major global health concern, impact millions of individuals every year [1,2]. The majority of these bacterial infections are those that can be transmitted through the food chain [3]. In 2015, the WHO presented the report on the estimates of the global burden of foodborne diseases, the first-ever estimates of the disease burden caused by 31 foodborne agents: bacteria, viruses, parasites, toxins, and chemicals at the global and sub-regional levels, which highlighted the possibility of over 600 million cases of foodborne illnesses and 420,000 deaths annually. According to the 2019 World Bank report on the economic impact of foodborne illnesses, the annual cost of treating foodborne illnesses is estimated to be USD 15 billion, and the total productivity loss linked to foodborne illness in low and middle-income countries is estimated to be USD 95.2 billion [4]. Despite modern surveillance and control systems, foodborne illnesses also continue to be a significant public health risk in higher-income countries. Recent estimates demonstrate that the economic impact of foodborne disease in the United States was roughly USD 75 billion by 2023, with deaths accounting for 56% and chronic consequences accounting for 31% of the overall cost, with bacterial pathogens contributing substantially to this burden [5]. Among foodborne bacterial pathogens, Pathogenic *E. coli*, particularly Shiga toxin-producing *E. coli* (STEC), is a leading cause of foodborne illness [6,7], which includes O157 and several non-O157 serotypes, is estimated to cause 2,801,000 acute infections, 3890 cases of hemolytic uremic syndrome (HUS), 270 cases of persistent end-stage renal disease (ESRD), and 230 deaths each year worldwide [8]. Although some strains of *E. coli* (K-12) are commonly found in the digestive tract of both humans and animals as a beneficial, non-pathogenic bacterium without causing any disease [9], some pathogenic strains such as STEC (O157:H7 and non-O157), EPEC, and EHEC have acquired some virulence factors that make them capable of causing disease [10,11] such as diarrhea. These pathogenic *E. coli*, which play an essential role in generating diarrheal illnesses, are classified into several pathotypes based on their virulence mechanisms [12,13]. The major diarrheagenic *E. coli* pathotypes include Enteroaggregative *E. coli* (EAEC), Enterotoxigenic *E. coli* (ETEC), Enteropathogenic *E. coli* (EPEC), and Shiga toxic *E. coli* (STEC), of which Enterohaemorrhagic *E. coli* (EHEC) is a subset. Because of its low infectious dose (less than 100 organisms) and dangerous nature, EHEC has been considered a particularly concerning zoonotic and foodborne illness, particularly in young children, the elderly, and those with impaired immune systems [14,15,16].

Among EHEC, *E. coli* O157:H7 is well known for being a serious foodborne pathogen and a primary cause of foodborne illnesses, including diarrhea and even death [17]. Most strains of EHEC have a plasmid that codes for a haemolysin and a chromosomally located locus of enterocyte effacement (LEE) pathogenicity island that enhances EHEC’s ability to cause intestinal injury and systemic complications by attaching bacteria tightly to host cells, which are hallmarks of attaching and effacing (A/E) lesions [18]. These genes that enable bacteria to create adhesion and effacing lesions in intestinal mucosa cells increase the severity of human infections. One of the most important and representative genes is the *eae* gene residing within the LEE pathogenicity island and encoding the intimin [19]. The *eae* gene is essential for the formation of attaching and effacing (A/E) lesions, which promote close adherence of *E. coli* O157:H7 to host intestinal epithelial cells. Through this attaching and effacing process, which is regulated by intimin protein, *E. coli* O157:H7 can adhere tightly to intestinal epithelial cells and cause illness [20]. The importance of the *eae* gene in the pathogenesis has prompted the use of the *eae* gene as a molecular diagnostic marker of pathogenic *E. coli* [21]. Nonetheless, the *eae* gene is highly genetically polymorphic and introduces a variety of intimin subtypes that may affect the virulence of the pathogen and the communication of this pathogen with tissues of the host [22]. Such variations, which are discovered by means of SNPs, insertions, or deletions, are important in host specificity [23,24] and tissue tropism [25,26,27], and provide a warning that close attention should be paid to these genetic alterations or evolutions in epidemiological studies and clinical diagnosis.

Conventional strategies for detecting genetic variations usually depend on sequencing techniques such as Sanger sequencing, which have many limitations, including high cost, lengthy processing times, and the need for sophisticated equipment [28]. Next-generation sequencing (NGS) [29] has become a high-throughput substitute for traditional sequencing techniques. However, it also requires sophisticated infrastructure and is prohibitively costly for many diagnostic laboratories. Of great significance, the resolution of the current NGS methods cannot be as precise as the single-nucleotide level. Other techniques, such as real-time PCR [30,31] using allele-specific probes and high-resolution melting (HRM) analysis [32], can provide faster results. Despite this, problems with affordability and availability persist especially in resource-constrained environments. Fluorescence In Situ Hybridization (FISH) is routinely employed to image RNA or genes at single-cell resolution [33]. However, it can only be used for in vivo imaging. Additionally, FISH has lower resolution and is difficult to use for screening small alterations, making single-nucleotide variant (SNV) identification nearly impossible [34]. The sensitivity of gene detection can be effectively improved by using nucleic acid amplification. The precise primer design could also be helpful for SNV identification. For example, Liu et al. reported the in situ loop-mediated isothermal amplification for detecting SNVs [35]. However, the complicated multiple primer sets design and the subsequent high probability of false-positive results greatly constrain the practical applications. Consequently, there is a growing need for straightforward, economical, and accessible methods that can be broadly utilized for easy and effective identification of SNPs in pathogenic bacteria. Among the available molecular approaches, a promising solution to this requirement is the ARMS-PCR [36]. This technique involves the design and application of allele-specific primers, which identify amplification on the basis of the consistency of the 3′ terminal base of the primer with the target DNA, and it leads to a high level of specificity in the identification of SNPs [37]. In addition, ARMS-PCR does not require any special high-value instruments since it can be performed in regular portable thermal cyclers. That is the reason for its affordable application in low-resource labs. Although it has these benefits, traditional ARMS-PCR detection usually relies heavily on agarose gel electrophoresis in order to visualize the final amplification products, which is tedious and complex [38]. To overcome this intrinsic drawback, the lateral flow strip (LFS) assay was designed and well combined with ARMS-PCR to facilitate the process of detection for providing direct readable output without the complexity of gel electrophoresis. LFS assays have the potential to provide direct judgment results with classic visualization, which is why they can be widely used for point-of-care tests, with the early pregnancy test strip as one of the most famous and typical popular home test products [39]. Such a kind of approach not only simplifies the whole workflow of the detection but also significantly reduces turnaround time for the final judgments [40].

This study aims to develop a simple, visual, and reliable method for rapid screening of genetic polymorphisms in the *eae* gene of *E. coli* O157:H7 at single-nucleotide resolution. We integrated ARMS-PCR with the LFS assay to deliver an intuitive visual readout of SNPs without requiring any advanced equipment. By targeting strain-specific SNPs in a key virulence gene, this approach offers a practical tool for rapid detection of pathogenic *E. coli* O157:H7, with potential applications in food safety surveillance and on-site testing.

## 2. Materials and Methods

### 2.1. Reagents and Instruments

All oligonucleotides, including the functionalized forward and reverse primer (amplified length 250 bp), 2XPCR Mix with blue dye, streptavidin (SAV), DNA marker (25–500 bp), and 4S Red Plus nucleic acid dye (1000×), HAuCl_4_, sucrose, Tween-20, bovine serum albumin (BSA), trisodium citrate and agarose, were purchased from Sangon Biotechnology Co., Ltd., Shanghai, China. Goat-anti-mouse secondary antibody (Anti-Ab), fluorescein isothiocyanate antibody (FITC Ab), and bovine serum albumin (BSA) were purchased from Baird Biotechnology Co., Ltd., Beijing, China. Lateral flow strip components including the absorbent pads, conjugate pads, sample pads, CN 95-nitrocellulose (NC) membrane and adhesive backing pads were all purchased from Jiening Biotechnology Co., Ltd., Shanghai, China. To confirm the specificity and sensitivity of specified primer sets and to optimize the detection conditions of the process, plasmids of the *eae* gene were synthesized by Sangon Biotechnology Co., Ltd., Shanghai, China.

### 2.2. Extraction of Genomic DNA and Sample Preparation for ARMS-PCR

Samples of milk and orange juice were collected from the local supermarket and confirmed to be free of *E. coli* contamination through the standard culture method [41]. DNA of mutant and wild-type *E. coli* was quantified and combined at specific proportions to produce various mutation ratios (100%, 50%, 25%, 10%, 5%, 1%, 0.1% and 0). The DNA combinations were then spiked into milk and juice to produce a complex dietary matrix. To mitigate the impact of food-derived matrix inhibition effects on PCR, DNA was extracted from each mixture utilizing a commercial TIANamp Bacteria DNA extraction kit (Provided by Tiangen Biotechnology Co. Ltd., Beijing, China) in accordance with the manufacturer’s guidelines. The liquid samples were diluted with the broth for enrichment and lysed directly for extraction. The extracted DNA was diluted in 50 μL of 1× TE buffer and utilized as a template for further analysis. To test the specificity of the ARMS-PCR technique, DNA from several common bacterial species was analyzed, including *Salmonella*, *Vibrio parahaemolyticus*, *Staphylococcus aureus*, *Pseudomonas aeruginosa*, and *Cronobacter sakazakii*, along with wild-type *E. coli*. Then, DNA was extracted using the same DNA extraction kit, the extracted genomic DNA was subjected to the ARMS-PCR experiment, and the results were finally observed using the lateral flow strip assay.

### 2.3. Preparation of AuNPs and AuNP-Labeled Anti-FITC Antibody Conjugates for LFS

The gold nanoparticles were prepared using the standard tri-sodium citrate reduction methodology with minimal alterations [42]. Overall, 2.55 mL of HAuCl_4_ (5 g/L) was mixed with 150 mL of deionized water in a flask. After that, the resulting solution was heated on a magnetic stirrer (IKA RCT basic stirrer with heating, IKA-Werke GmbH & Co. KG, Staufen, Germany) to boiling while being stirred magnetically at 1000 rpm. Following that, 2.25 mL of trisodium citrate solution (1%) was added quickly to react with HAuCl_4_, causing the solution’s color to change from black to gray, then to a stable wine-red. Ultimately, the resulting AuNP solution was stirred and allowed to cool to room temperature and kept at 4 °C in the refrigerator before using. For preparation of the AuNP/FITC-Ab conjugates, the pH of AuNPs was first adjusted with 10 μL K_2_CO_3_ (0.1 M), and then 4 μL FITC-Ab (1 mg/mL, dissolved by 10 mM PB buffer) was added. After incubating at room temperature for 1 h, 100 μL 10% BSA was added to block the residual active sites on AuNPs for 60 min to prevent non-specific adsorption. After that, the AuNP/FITC-Ab conjugates were concentrated by freezing (4 °C) centrifugation at 9500 r/min for 10 min using DLAB D3024R high-speed refrigerated centrifuge machine (DLAB Scientific Co., Ltd., Beijing, China). For the subsequent LFS assembly, the precipitate was resuspended in 100 μL Resuspension E buffer (1 mM Tris-HCl, 1% BSA, 0.25% PEG 20000, 10% sucrose), which was then sprayed onto the conjugation pad (6 × 300 mm) by the Bio-Dot sprayers (Irvine, CA, USA), and then allowed to dry overnight at 27 °C.

### 2.4. Assembly of LFS

The LFS is made up of a PVC back-plastic plate (60 × 300 mm), an absorbent pad (18 × 300 mm), an NC membrane (25 × 300 mm), a conjugation pad (6 × 300 mm), and a sample pad (18 × 300 mm). The buffer solution (pH 8.0) containing 200 mL ddH_2_O, 50 mM Tris-HCl, 0.15 mM NaCl, and 0.25% Triton-100 was used to treat the sample pad, while the solution (pH 8.0) containing 100 mL PB buffer, 5% sucrose, 2.5% PEG-20000, 1% alginate, and 0.3% Tween-20 was used to pretreat the conjugation pad. After that, both the conjugation pad and sample pad were dried in a drying oven (Jingqi DHG-9073A incubator oven) provided by Shanghai Jingqi Instrument Co., Ltd., Shanghai, China at 27 °C overnight. Using a Bio-Dot spraying device (Irvine, CA, USA), the NC membrane was sprayed with 20 μL SAV (1 mg/mL, dissolved by 10 mM PB buffer) and 20 μL second anti-mouse Ab (1 mg/mL, dissolved by 10 mM PB buffer) at a rate of 0.5 μL/cm to prepare the test line (T line) and the control line (C line), respectively. Following these pretreatments, the LFS was assembled by overlapping the sample pad, conjugation pad, NC membrane, and absorbent pad on the back plastic plate with overlap of 2 mm with the neighbor pad. Finally, the LFS was sliced into 3 mm width using an automatic strip cutter (Irvine, CA, USA).

### 2.5. Primer Design for the Accurate Identification of SNPs in the Target eae Gene

The primers used in this study were designed in accordance with the ARMS-PCR amplification principle using Primer Premier 6.0 and synthesized by the Sangon Biotechnology Co., Ltd., Shanghai, China. The detailed sequence information of primer and probe is given in Table A1. The concept behind the design of ARMS-PCR primers is that, when the target DNA is normal, a single-nucleotide mismatch at the 3’-OH end of primer prevents Taq DNA polymerase from extending the primer under the proper PCR conditions, resulting in only the one-base-mutated targets being amplified. To improve the ability to differentiate the normal and mutant alleles, an intentional mismatch was also introduced at the third site of ARMS primer.

NCBI primer blast http://www.ncbi.nlm.nih.gov/tools/primer-blast/, accessed on 2 April 2025) was used to check the specificity to other potential templates. The online Oligo Evaluator program (http://www.oligoevaluator.com, accessed on 2 April 2025) was used to examine the suspected primer-dimer and hairpin structures.

### 2.6. ARMS-qPCR for Primer Design Evaluation

To identify the most effective primer sets for distinguishing the wild and mutant alleles, allele-specific real-time PCR (ARMS-PCR) was performed using a Bio-Rad CFX96 system (Bio-Rad Laboratories, Hercules, CA, USA). Each 25 µL reaction system contained 12.5 µL qPCR master mix, 0.7 µL (10 µM) of forward and reverse primer, 1.2 µL (10 µM) TaqMan probe, 10.5 μL of H_2_O and 1 µL template DNA.

### 2.7. ARMS-PCR Assisted Rapid and Visual Identification of SNP in eae Gene of E. coli with the Lateral Flow Strip

Primers that demonstrated the highest discrimination capability between wild-type and single-nucleotide mutant alleles in *eae* gene with ARMS-qPCR were designed and optimized for additional verification using conventional ARMS-PCR. ARMS-PCR amplification was performed in a 25 μL volume containing 0.5 μL of primer sets (10 μM) 1 μL of DNA templates, 12.5 μL of 2 × sanTaq PCR Mix, and 10.5 μL H_2_O with the following amplification conditions: initial denaturation at 94 °C for 3 min, followed by 30 cycles of denaturation at 94 °C for 45 s, annealing at 60 °C for 45 s, and extension at 72 °C for 1 min, with an additional extension step at 72 °C for 5 min. For the convenient visual identification of SNPs in the *eae* gene of *E. coli*, both the forward and reverse primers were modified with FITC and biotin at the 5’ end, respectively. Subsequently, the amplicons would be dual-labeled with both FITC and biotin in the amplification, which would be measured by the designed lateral flow strip.

### 2.8. Image Analysis

The quantitative optical intensity of the T line was quantified using ImageJ Software version 1.53c, National Institute of Health, Bethesda, MD, USA. A fixed-size rectangular region of interest (ROI) was selected to cover the T line, and the ROI’s integrated density value was used to calculate T line intensity. These values were plotted against mutation proportions to generate signal intensity-mutation proportion curves. The control (C) line confirmed the validity of each test. All images were captured under consistent lighting, camera settings, and imaging conditions to ensure comparability.

## 3. Results

### 3.1. Detection of Mutation in eae Gene of E. coli by the Designed ARMS-PCR Mediated Lateral Flow Strip in the Visual Mode

**Figure 1:**
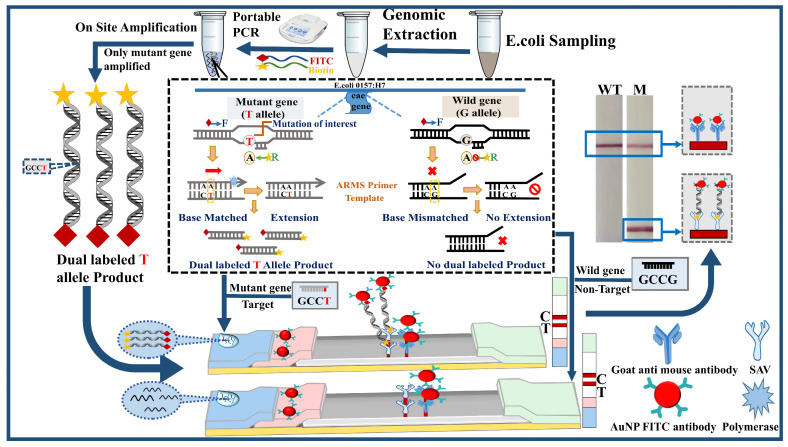
Schematic illustration of ARMS-PCR combined with LFS for distinguishing between wild-type and Mutant gene of *E. coli* O157:H7 with single-nucleotide resolution.

Rapid identification of SNPs in the *eae* gene of *E. coli* enables the differentiation of potentially dangerous pathogenic strains and provides insight into genetic variants associated with virulence factors. As illustrated in Figure 1, the mutant-specific primer designed with several mismatched nucleotides at its 3’ end decreases amplification efficiency for the wild-type template due to poor binding affinity. The ARMS assay demonstrated high sensitivity and specificity, enabling precise discrimination between matched and single-nucleotide mismatched targets and reliable identification of the mutated target at the visible detection limit. As shown in Figure 1, with the help of two labeled primers, DNA amplification products are simultaneously labeled at one end with FITC and at the other end with biotin. And further integrating with the lateral flow strip can lead to the rapid visible identification of target SNPs. Typically, in the presence of the target mutated DNA in the sample, it will bind with the AuNP-modified FITC antibody and the pre-immobilized SAV on the T line of LFS to form the AuNP-amplicon-SAV structure on the T line, retaining the AuNPs on the T line and producing the optical signal on the T line. On the contrary, with the wild type or the absence of the mutated genes, no dual-labeled amplicons will be formed, the AuNP-anti-FITC will not be retained on the T line, and no signal can be observed. And under any conditions, the AuNP-anti-FITC could be recognized by the second anti-mouse antibody on the C line, showing optical signals on the C line and indicating the validity of this detection. With this ARMS-PCR-mediated LFS, single-nucleotide variation in the target gene could be easily monitored in the visible observation mode, providing the direct results and information for timely response and effective treatment of the mutated strains of *E. coli*.

### 3.2. Possibility Verification of the Designed ARMS-PCR-Mediated LFS Assay for Precise Visual SNP Identification

**Figure 2:**
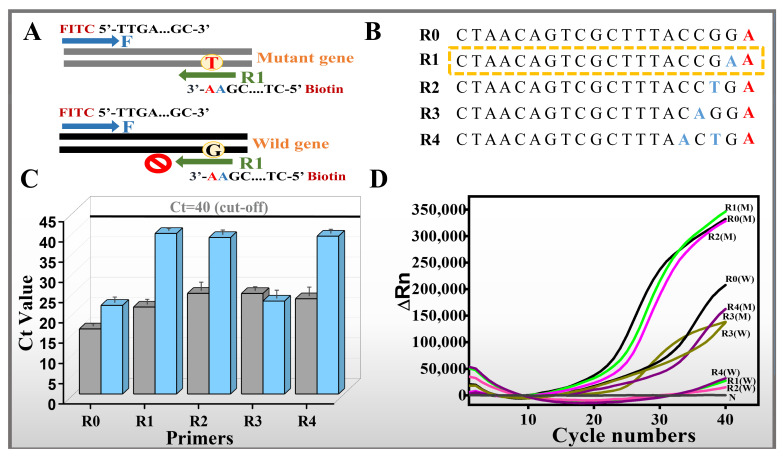
ARMS-PCR primer optimization: (**A**) ARMS-PCR assay methodology for identifying wild-type (WT) and mutant (MT) DNA sequences. (**B**) Nucleotide sequence of the R primer. Mismatched base locations are marked in red. (**C**) Comparison of Ct values for mutant (MT) and wild-type (WT) genes gray and blue bar respectively. (**D**) qPCR curves of WT and MT genes using different R primers.

**Figure A2:**
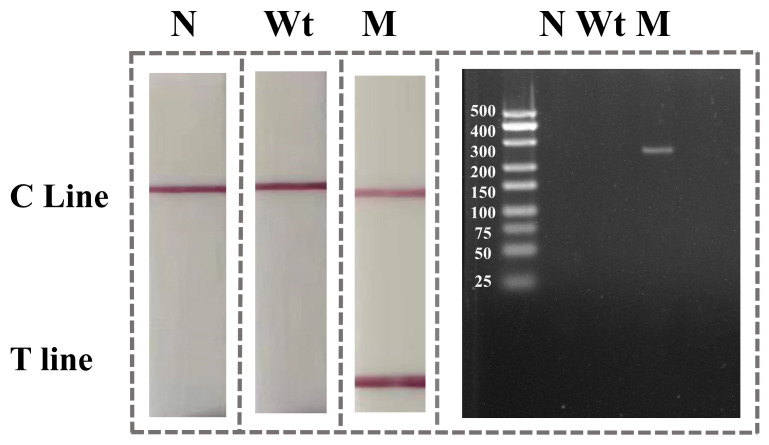
Detection of mutant allele by ARMS-PCR followed by LFS: LFS result, Lane N: Negative; Lane Wt: Wild gene; Lane M: Mutant gene; T: Test line; C: Control line. Band in Lane M shows amplification of mutant gene. Gel electrophoresis, mutant sample displaying 250 bp band confirms successful amplification of mutant allele.

The main purpose of this assay is to realize rapid and easy identification of SNPs within the *eae* gene of *E. coli* O157:H7 in the visible mode. In general, it is exceedingly challenging to differentiate alleles with solely PCR primers featuring a single-nucleotide alteration at the 3′-end. Therefore, by introducing an additional mismatch at a different position from the 3′-terminus in the *eae* allele-specific reverse primers, the specificity of the SNP differentiation may be further improved and guaranteed especially in the low mutation abundance. To verify the performance of the different designed primers in SNP identification, the wild-type and the mutated-type genes were first tested with the real-time PCR. From the real-time PCR results in Figure 2, primers R1, R2 and R4 did not amplify wild-type genes (with Ct >40), indicating the good specificity for identification of SNP mutated-type genes, while the primers R0 and R3 produced positive signals for both wild-type and mutated-type genes, showing no differentiation between the wild-type and mutated genes. Furthermore, in detail, among primers R1, R2, and R4, primer R1 had the best amplification effect for just the mutated-type gene. Therefore, the designed R1 was adopted for further visible identification research. Meanwhile, both the agarose gel electrophoresis and lateral flow strip results demonstrated that only the mutant-type gene had the characteristic band of 250 bp in the gel and the positive signal on the T line of LFS, further indicating the technical feasibility of the designed ARMS-PCR mediated LFS platform for rapid and visible identification of SNP in *eae* genes of *E. coli* O157:H7 *(*Figure A2).

### 3.3. Visual-Identification Performance of the SNP in the Mutant Genes with the Designed ARMS-PCR-LFS

**Figure 3:**
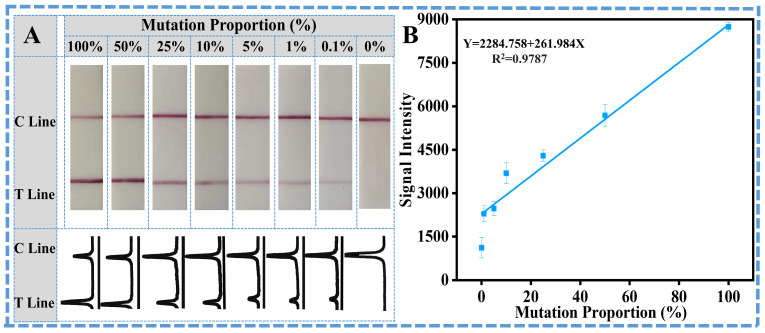
Authentication performance of ARMS-PCR LFS: (**A**) Sensitivity evaluation using different concentrations of mutant DNA. (**B**) Linear correlation between T line signal intensity and *E. coli* concentration.

**Figure 4:**
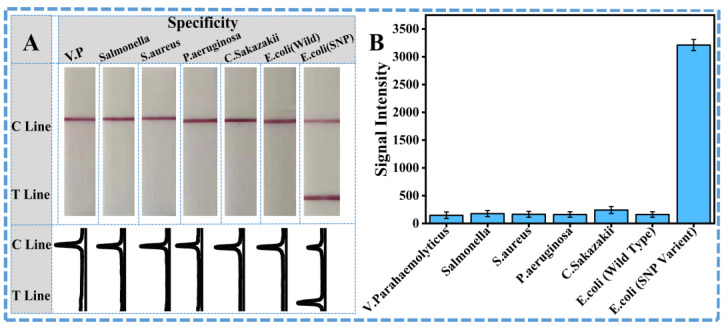
Selectivity assay results of amplification-assisted molecular LFS: (**A**) Visual observation result of amplification-assisted molecular LFS and corresponding curves of ImageJ-treated results. (**B**) Quantification of T line intensity induced by different analytes using software ImageJ.

After verifying the technical feasibility for rapid identification of SNPs in the *eae* genes, some critical parameters of PCR were optimized to achieve the best amplification effect with the highest identification efficiency for on-site applications. An amount of 0.5 µL 10 µM primer for each reaction was adopted for the specific amplification. And the annealing temperature of 60 °C was determined as optimal for the balance between specificity and signal strength of the target band. To get the most efficient amplification without any non-specific products, 30 cycles were adopted as the best condition to integrate with the lateral flow strip. The mutant-type gene was spiked into the wild-type gene to prepare the samples with various mutation ratios (from 0 to 100%), and then the samples were all detected with the developed ARMS-PCR mediated LFS. From the results in Figure 3, it could be observed that, with the increase in mutant ratio from 0 to 100%, the signal on the T line of LFS is increased accordingly. For visual qualitative analysis, the limit of detection (LOD), 0.1%, was defined as the lowest concentration of mutated DNA at which the T line could be seen clearly with the naked eye. To ensure uniformity, all observations were carried out under controlled lighting conditions, with lateral flow strips set on a neutral white background at a predetermined distance from the observers. Each test was reviewed independently by two observers, and the LOD was only documented when both observers could clearly see the T line. A test was considered positive (“Yes”) when the T line was clearly visible, showing the presence of the mutant DNA, whereas a negative result (“No”) was recorded when the T line was absent, reflecting the absence of mutant DNA or the mutated ratio lower than 0.1%. This visual LOD serves as a practical reference for yes/no detection, which was further analyzed by ImageJ software, allowing more accurate semi-quantitative estimation of the T line signals. As shown in Figure 3B, the calibration curve is also constructed based on the quantitative signals on the T line of LFS, and the quantitative detection limit of the mutation ratio could be as low as 0.1% according to the 3σ criterion. With this designed primer set and the ARMS-PCR mediated LFS, the specificity against other common non-target bacteria, including *Salmonella*, *Vibrio parahaemolyticus*, *Staphylococcus aureus*, *Pseudomonas aeruginosa*, and *Cronobacter sakazakii*, was also considered. Results in Figure 4A,B well demonstrate that other common non-target bacteria and the wild-type *E. coli* O157:H7 with the *eae* gene could not induce the signals on the T line, while only the mutation-type *E. coli* O157:H7 could induce the obvious observable signal on the T line of LFS. All these results have strongly shown the satisfactory detection performance and specificity for the SNP gene samples.

### 3.4. Practical Detection Research of SNP Mutated eae Genes of E. coli in Spiked Wild-Type E. coli Samples

**Figure 5:**
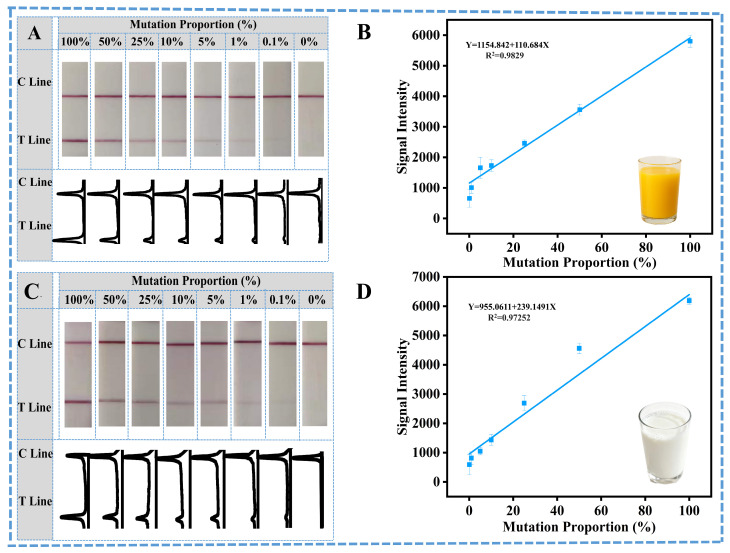
Detection of *E. coli* in real food samples: (**A**) Sensitivity evaluation using juice samples spiked with different ratios of mutant DNA and corresponding curves of ImageJ-treated results. (**B**) Linear correlation between T line signal intensity and *E. coli* concentration in juice sample. (**C**) Sensitivity evaluation using Milk samples spiked with different ratios of mutant DNA and corresponding curves of ImageJ-treated results. (**D**) Linear correlation between T line signal intensity and *E. coli* concentration in Milk sample.

Finally, to further verify the practical application performance of this ARMS-PCR mediated LFS, the mutated *eae* gene of *E. coli* was spiked into the samples with different matrices. Then, all samples were detected with the ARMS-PCR mediated LFS, and the results are shown in Figure 5. In detail, all spiked mutated *eae* genes could be well detected, and the corresponding calibration curves could be constructed for quantitative analysis for different sample matrices. Comparatively, the detection results of the spiked samples could come to the same conclusion as the standard gene samples. All these results well demonstrate the excellent capability of this ARMS-PCR mediated LFS for rapid and easy identification of SNPs in the visible mode.

The developed ARMS-PCR-LFS has been constructed for rapid and easy identification of SNPs in *eae* genes of *E. coli* O157:H7 in the visible mode without any additional high-value instruments, which is suitable for on-site or resource-limited settings. The traditional complicated SNP assay is converted into a binary presence/absence signal with the designed ARMS-PCR mediated LFS, highlighting the potential of ARMS-PCR mediated LFS as a powerful alternative for SNP assay. The whole operation process and the final result judgment are also very simple even for non-professional personnel, with the presence of the T line on the LFS indicating the occurrence of SNPs in the target genes. And the cost of each identification is also acceptable for various scenarios with each SNP identification costing less than USD 1. However, to realize the rapid high-throughput screening of potential evolution or mutation of target pathogens for epidemic or clinically precise therapy, simultaneous multiplex screening of SNPs at different sites is of great importance. And to further simplify and avoid the dependence of the device, the isothermal amplification should be adopted for the replacement of PCR. Related research is still ongoing in our lab for practical applications.

## 4. Conclusions

In this study, the single-nucleotide polymorphism (SNP) in the *eae* gene of *E. coli* O157:H7 was rapidly and visibly identified by using the designed ARMS-PCR mediated LFS method. With the designed primer set, the difference in a single nucleotide in the target genes could be precisely identified. And the identification of SNPs could be converted into a simple, visible, yes/no outcome on the T line of the LFS without any sophisticated instrumentation. The SNPs in the target *eae* gene have been simply judged: the presence of the T line indicates the occurrence of SNPs in the target genes, while only the C line of the LFS indicates no SNPs in the specific site of the genes of *E. coli* O157:H7, making it an ideal candidate for point-of-care and field-deployable SNP diagnostics. The single-nucleotide-mutated genes have been well identified with the mutation ratio as low as 0.1% by observation and quantitative analysis in even food samples with complicated matrices, strongly demonstrating the great potential for rapid and on-site identification of SNPs in the genes of interest. Multiplex screening of different mutated sites and integration with isothermal amplifications will further widen this designed SNP identification platform for practical applications in the surveillance of pathogen evolution, and related research is ongoing in our lab.

## Figures and Tables

**Figure A1:**
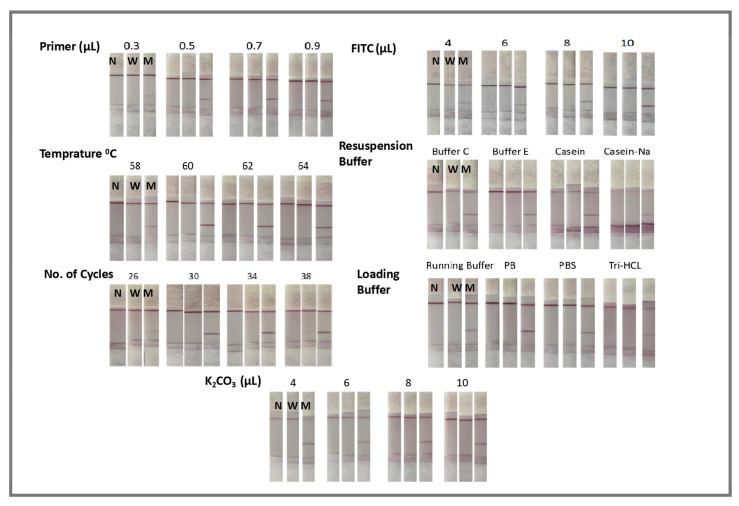
Optimization conditions of ARMS-PCR-LFS: primer concentration, amplification temperature, number of cycles, loading buffer (Running buffer, PB, PBS, Tris-HCL), volume of K_2_CO_3_ (0.1 M), type of resuspension buffer (Buffer C (10 mM PB, 1% BSA, 0.25% Tween-20, 10% Sucrose), Buffer E (1 mM Tris-HCl, 1% BSA, 0.25% PEG 20000, 10% sucrose), Casein, Casein-Na), Volume of FITC-Ab.

## Data Availability

The data presented in this study are available upon request from the corresponding author due to the intellectual property.

## Appendix A

### Appendix A.1

**Table A1:** Primers and probe sequences used to detect SNPs of *eae* gene.

Primers and Probe	Primer and Probe Sequence	Size (bp)	Method
F—PCR R1	TTGATCAAACCAAGGCCAGC CTAACAGTCGCTTTACCGAA	250	ARMS-PCR
F—LFS R1	FITC-TTGATCAAACCAAGGCCAGC Biotin-CTAACAGTCGCTTTACCGAA	250	ARMS-PCR-LFS
F—qPCR R1	TTGATCAAACCAAGGCCAGC CTAACAGTCGCTTTACCGAA	250	ARMS-qPCR
P—qPCR	5’-FAMTCCCGTGGTTGCTTGCGTTTGAGACT-BHQ1		

F: Forward Primer; R: Reverse Primer; and P: Probe.

### Appendix A.2. Optimization Experimental Conditions of ARMS-PCR-LFS

We optimized the experimental conditions for LFS to identify the target gene. Primer (0.3 μL, 0.5 μL, 0.7 μL, 0.9 μL), Temperature (58 °C, 60 °C, 62 °C, 64 °C), Cycle numbers (26, 30, 34, 38), FITC-Ab, K2CO3, Type of loading buffer (Running buffer, PB, PBS, Tris-HCL), and Type of Resuspension Buffer (Buffer C, Buffer E, Casein, Casein-Na) were optimized. The optimal conditions determined for each parameter, as shown in Figure A1 (primer 0.5 μL, annealing Temperature 60 °C, 30 cycles, FITC 4 μL, K_2_CO_3_ 10 μL (0.1M), Loading Buffer PBS, Resuspension Buffer E), were used for all subsequent experiments.
